# The inhibitory effects of AR/miR-190a/YB-1 negative feedback loop on prostate cancer and underlying mechanism

**DOI:** 10.1038/srep13528

**Published:** 2015-08-28

**Authors:** Shaohua Xu, Tao Wang, Wen Song, Tao Jiang, Feng Zhang, Yu Yin, Shi-Wen Jiang, Kongming Wu, Zuoren Yu, Chenguang Wang, Ke Chen

**Affiliations:** 1 Department of Gynecology, Shanghai First Matenity and Infant Hospital, Tongji University School of Medicine, Shanghai, China; 2 Department of Urology, Tongji Hospital, Tongji Medical College, Huazhong University of Science and Technology, Wuhan, China; 3 Cancer Center, Union Hospital, Tongji Medical College, Huazhong University of Science and Technology, Wuhan, China; 4 Department of General Surgery, QingZhou People Hospital, QinZhou, China; 5 Department of Gastrointestinal Surgery, The Third Affiliated Hospital of Soochow University, Changzhou, China; 6 Department of Pathology, Anhui Medical University, Hefei, China; 7 Department of Biomedical Science, Mercer University School of Medicine, Savannah, GA, USA; 8 Department of Oncology, Tongji Hospital, Tongji Medical College, Huazhong University of Science and Technology, Wuhan, China; 9 Research Center for Translational Medicine, Shanghai East Hospital, Tongji University School of Medicine, Shanghai, China; 10 Key Laboratory of Tianjin Radiation and Molecular Nuclear Medicine; Institute of Radiation Medicine, Peking Union Medical College & Chinese Academy of Medical Sciences, Tianjin, China

## Abstract

Prostate cancer at advanced stages including metastatic and castration-resistant cancer remains incurable due to the lack of effective therapies. MiR-190a belongs to the small noncoding RNA family and has an important role in breast cancer metastasis. However, it is still unknown whether miR-190a plays a role in prostate cancer development. Herein, we first observed AR/miR-190a/YB-1 forms an auto-regulatory negative feedback loop in prostate cancer: miR-190a expression was down-regulated by AR activation; YB-1 functions are as an AR activator; miR-190a inhibited AR expression and transactivation through direct binding to 3′UTR of *YB-1* gene. MiR-190a contributes the human prostate cancer cell growth through AR-dependent signaling. Moreover, we examined the expression of miR-190a and observed a significant decrease in human prostate cancers. Reduced expression of miR-190a was inversely correlated to AR levels of prostate cancer patients, and patients with higher miR-190a expression in their tumor have improved tumor-free survival. Taken together, our findings identified a biochemical and functional link between miR-190a with reduced expression in advanced prostate cancer, YB-1 and AR signaling in prostate cancer.

Prostate cancer is one of the most common malignancies in men in western world, accounting for about 240,000 new cases and 29,000 deaths in the United Stated in 2013^1^. The progression of prostate cancer normally starts from castration-sensitive, castration-resistant, inevitably developing into highly metastatic disease^2^. Androgen and androgen receptor (AR) are critical effectors of prostate cancer^3^,^4^. Consequently, androgen deprivation therapy is typically employed as a first-line treatment for prostate cancer patients, while initial responses are generally positive, prostate tumors frequently recur and progress to a lethal form known as castration-resistant prostate cancer (CRPC), associate with poor prognosis and low survival rates^4^,^5^.

Studies have shown that a class of small non-coding RNAs (miRNA) plays a pivotal role in prostate cancer by acting as oncogene or tumor suppressor^6^,^7^. However, little known about the functional interaction between miRNA expression and androgen/AR signaling. MiR-190a belongs to the miRNA family and is located in the tail intron regions of two genes on 15q22.2. While miR-190a is downregulated in aggressive neuroblastoma (NBL), its overexpression leads to the inhibition of tumor growth and prolonged dormancy periods in fast growing tumors^8^. A recent study showed that miR-190a is involved in estrogen receptor (ERα) signaling, causing inhibition of breast tumor metastasis^9^. It is currently unknown whether miR-190a plays any role in prostate cancer development.

In this study, we observed inverse correlation of miR-190a expression and PSA levels in prostate cancer, where patients with higher miR-190a expression in their tumors had improved disease-free survival. In human prostate cancer cells, AR/miR-190a/YB-1 signaling forms an auto-regulatory negative feedback loop, where miR-190a contributes to the human prostate cancer cell growth through AR-dependent signaling. Taken together, our findings reveal a tumor suppressive mechanism for AR/miR-190a/YB-1 negative feedback loop in prostate cancer progression.

## Results

### MiR-190a expression is repressed by AR upon androgen treatment

**Figure 1:**
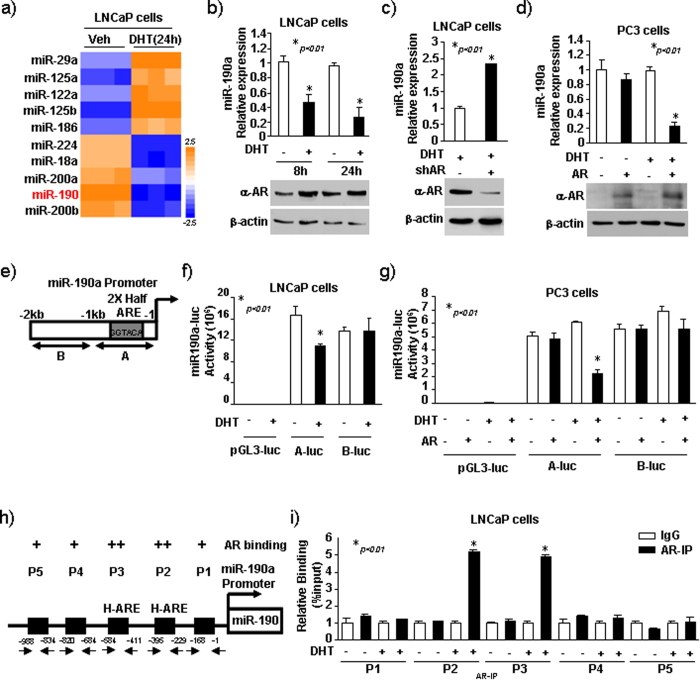
MiR-190a expression is repressed by AR upon androgen treatment. (**a**) miRNA array analysis showed that miRNAs were differentially expressed in LNCaP and LNCaP/DHT. (**b**) LNCaP cells treated with 10 nm dihydrotestosterone (DHT) for 8–16 hrs with the total RNA was isolated. miR-190a expression by qRT-PCR. (**c**) miR-190a expression by qRT-PCR in LNCaP cells with stably knockdown of AR. LNCaP cells treated with 10 nM DHT for 16 hrs. (**d**) miR-190a expression determined by qRT-PCR in PC3 cells with stably overexpression of AR. PC3 cells treated with 10 nM DHT for 16 hrs. (**e–g**) miR-190a promoter reporters (−9 to −1053, −1121 to −2013) assessed in LNCaP and PC3 cells. DHT repressed activity of miR-190a gene reporters ((−9 to −1053) in the presence of AR. (**h,i**) CHIP analysis of AR for miR-190a promoter region in LNCaP cells. LNCaP cells treated with DHT or vehicle for 16 hrs. CHIP assay was performed using an anti-AR antibody.

To determine the crucial miRNAs involved in prostate cancer progression, we first performed Cancer micro-RNA Array that contains 95 different miRNAs in LNCaP and LNCaP/DHT cells. Differentially expressed miRNAs with at least 2-fold alternation were selected (Fig. 1a). Consistent with other studies, we also found that miR-200a, miR-200b, miR-18a and miR-224 were among the top 10 downregulated miRNAs, while miR-29, miR-125a, miR-125b, miR-122 and miR-186 were upregulated in LNCaP/DHT compared with LNCaP. Importantly, miR-190a, a tumor suppressor miRNA, was newly identified miRNA that was remarkably downregulated in LNCaP/DHT compared with LNCaP. To further validate this finding, LNCaP cells were treated with 10 nM dihydrotestosterone (DHT) for 8–16 h and total RNA was extracted. As shown in Fig. 1b, qRT-PCR analysis revealed that miR-190a levels were decreased upon androgen treatment in LNCaP cells. In order to confirm whether the effect of androgen is through AR, we modulated the levels of AR in LNCaP and PC3 cell lines by transfecting them with either shRNA or a vector encoding AR followed by DHT treatment. As shown in Fig. 1c,d, shRNA-mediated knockdown of AR in LNCaP cells induced increase in miR-190a levels, whereas overexpression of AR in PC3 cells significantly reduced miR-190a levels. Collectively, these observations indicate that miR-190a is downregulated by AR activation.

Androgen is known to regulate gene expression through binding to AR, which then binds to androgen response element (ARE) in the promoter region of AR target genes. We analyzed the region between 2,000-bp and 1-bp upstream of miR-190a promoter and identified two putative half site for ARE (Fig. 1e). In order to evaluate the effects of androgen on miR-190a transcription rate, we performed luciferase reporter assays. The promoter region of miR-190a was cloned upstream of luciferase coding sequence and two separate clones (A-luc: −9 to −1053 containing the ARE; B-luc: −1121 to −2013 lacking ARE) were obtained. The luciferase activity was examined in AR-positive LNCaP cells and AR-negative PC3 cells. As shown in Fig. 1f, the LNCaP cells transfected with A-luc (containing ARE) showed lower luciferase activity in the presence of DHT, while cells transfected with B-luc showed no response to DHT treatment. However, no repression was seen in PC3 cells transfected with A-luc (Fig. 1g), while the overexpression of AR in PC3 cells reduced A-luc reporter activity by 50% following DHT treatment (Fig. 1g). This result showed androgen/AR-dependent repression of miR-190a transcription functions through the promoter of miR-190a gene. In order to further determine whether identified half-ARE within miR-190a gene promoter is involved in the repression, we conducted chromatin a immunoprecipitation (ChIP) assay. ChIP assay was performed in LNCaP cells with or without DHT treatment using PCR primers covering the sequence of interest (Fig. 1h). As shown in Fig. 1i, AR was detected in the P2 (−395 to −229) and P3 (−584 to −411) regions of miR-190a promoter with an increased association to the promoter in the presence of DHT. Taken together, these results suggest that androgen inhibits miR-190a expression through direct binding to the half-site of ARE in miR-190a promoter.

### MiR-190a inhibits AR expression and transactivation

Next, we analyzed the levels of miR-190a in several most commonly used prostate cell lines. miR-190a expression in AR-positive cell lines was detected at lower levels (LNCaP, C4-2, LAPC4, 22Rv1) when compared to AR-negative cell lines (RWPE1, PC3, DU145) (Supplemental Fig. 1a,b). These results suggest a functional interaction between miR-190a and AR during prostate cancer progression.

**Figure 2:**
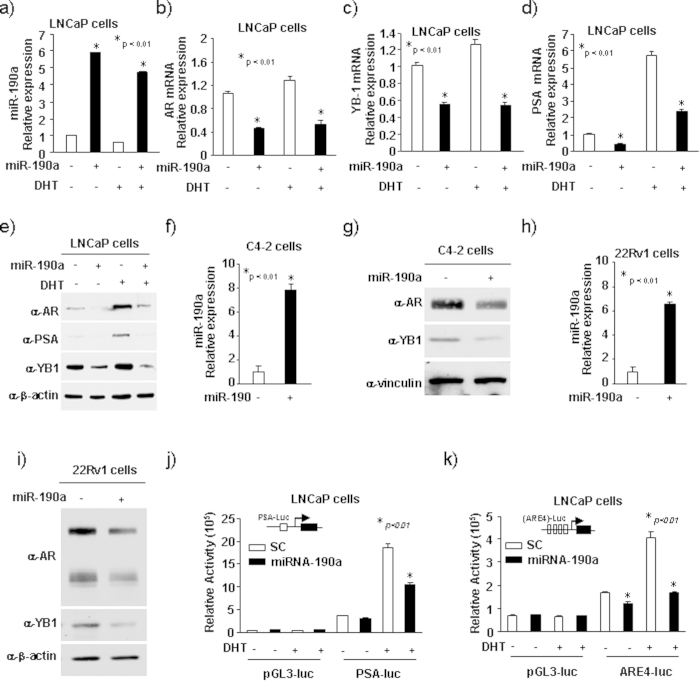
MiR-190a inhibits AR expression and its trans-activation. (**a–d**) MiR-190a, AR, YB-1 and PSA mRNA levels determined by qRT-PCR in LNCaP cells with stable overexpression of miR-190a. LNCaP cells were treated with 10 nM DHT for 16 hrs. (**e**) AR, YB-1 PSA protein levels determined by Western blot in LNCaP cells with stable overexpression of miR-190a. LNCaP cells treated with 10 nM DHT for 16 hrs. (**f–i**) AR, YB-1 protein levels were determined by Western blot in C4-2 and 22Rv1 cells with overexpression of miR-190a. (**j,k**) Androgen-responsive luciferase reporter genes (PSA-Luc, ARE4-Luc) assessed for AR activity. LNCaP cells with miR-190a overexpression were treated with DHT for 16 hrs.

In order to determine whether miR-190a regulates AR signaling, we established stable cell lines (LNCaP, C4-2, and 22Rv1) through lentiviral transduction. As shown in Fig. 2a,f,h, the expression of miR-190a in these cell lines were higher than in scramble control cells. First, we detected the effect of overexpression miR-190a on androgen signaling in LNCaP cells. Since YB-1 is known to be upregulated during prostate tumor progression, and found to promote AR expression^10^, qRT-PCR and Western blot analyses were used to show the significant decrease in mRNA and protein levels of AR, YB-1 and PSA upon androgen treatment (Fig. 2b–e). Similarly, overexpression of miR190 in C4-2 and 22Rv1 cells decreased the protein levels of AR and YB-1 (Fig. 2g,i).

### YB-1 Functions as an AR activator in prostate cancer

**Figure 3:**
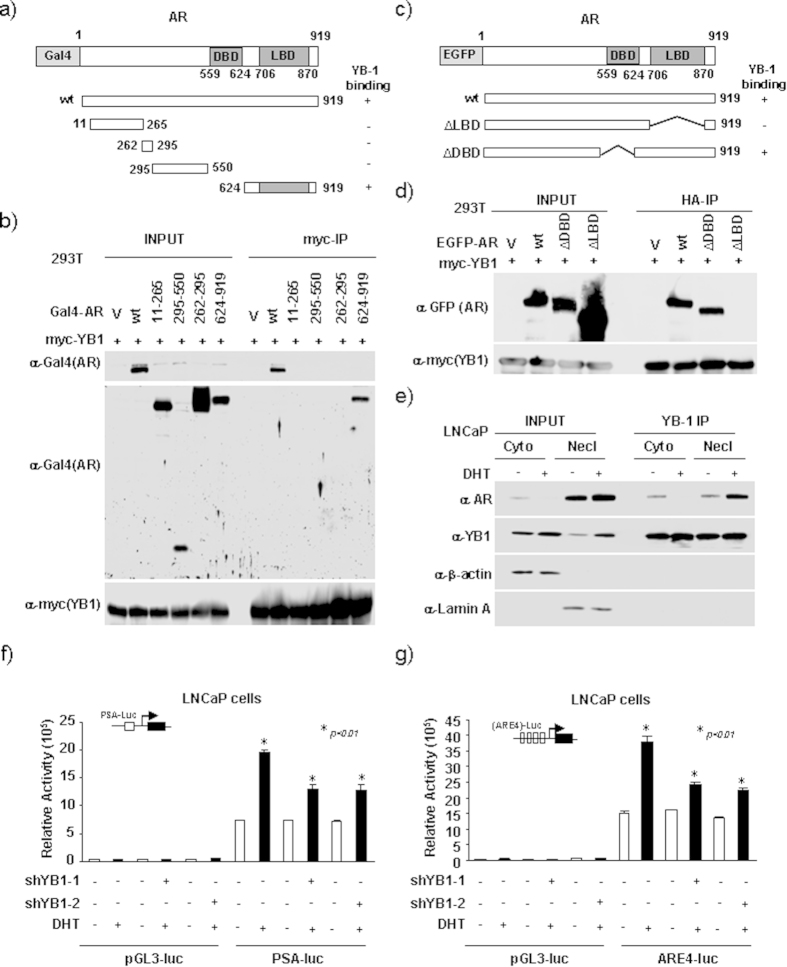
YB-1 Functions as a novel AR activator in prostate cancer. (**a**) Schematic representation of YB-1, AR, and AR mutation expression vectors. (**b**) Immunopercipitation-Western blot analysis was conducted using 293 T cells transfected with expression vectors encoding either N-terminal myc-tagged YB-1 or Gal4-tagged AR expression vectors. (**c**) Schematic representation of YB-1 and AR mutation expression vectors. (**d**) Immunopercipitation-Western blot analysis conducted of 293 T cells transfected with expression vectors encoding either N-terminal myc-tagged YB-1 or EGFP-tagged AR mutation expression vectors. All the data are representative of N = 3 separate experiments. (**e**) IP-Western blotting in LNCaP cells cells treated with 10 nM DHT. IP was conducted with an YB-1 antibody against endogenous YB-1. Subcellular fractionation of cells was performed as described. (**f,g**) Androgen-responsive luciferase reporter genes (PSA-Luc, ARE4-Luc) were assessed for AR activity. LNCaP cells with YB-1 knockdown were treated with 10 nM DHT.

A previous study showed how YB-1 promotes AR transcription via binding to the Y-box in the AR promoter region^10^, while we first examined the interaction between YB-1 and AR at the protein level. A series of Gal4-AR mutant (residues 11–265, 262–295, 295–550 and 624–919) expression vectors were transfected into 293 T cells (Fig. 3a) with the N-terminal c-myc epitope used for immunoprecipitation of YB-1. Transfection of 293 T cells with a series of AR mutants showed association of AR and YB-1. AR-LBD (ligand binding domain, residues 624–919) was proved to be sufficient for binding to YB-1 (Fig. 3b). To further confirm that YB-1 only bound to AR-LBD, EGFP-AR-∆DBD (deletion of DNA-binding domain) and EGFP-AR-∆LBD (deletion of LBD) expression vectors were transfected into 293 T cells (Fig. 3c). The N-terminal myc epitope used to immunoprecipitate YB-1 confirmed that, the deletion of the AR-LBD abolished binding toYB-1, shown in Fig. 3d. These studies indicate that AR-LBD is required for binding YB-1.

When AR is activated by the endogenous androgenic ligands, the ligand-receptor complex translocates from cytoplasm into the nucleus, and in association with co-regulatory factors, binds to specific androgen responsive elements in the regulatory regions of AR target genes^11^. In general, AR agonist modulates AR function by binding to the AR-LBD^12^. Previous studies showed nuclear YB-1 expression significantly correlated with the Gleason score and AR expression in prostate cancer tissues^10^. In order to determine whether endogenous YB-1 associates with AR in the presence of ligand, we performed IP Western blot analyses with separate cytoplasmic and nuclear fractions of proteins. An antibody directed toward endogenous YB-1 coprecipitated AR in LNCaP cells treated with DHT. As shown in Fig. 3e, DHT increased the association of YB-1 with AR in the nucleus, while it decreased the binding of YB-1 to AR in the cytoplasm. Next, we conducted PSA-luc and ARE4-luc luciferase reporter assays to evaluate the effects of YB-1 on AR transactivation. As shown in Fig. 3f,g, knockdown YB-1 in LNCaP cells reduced DHT-induced PSA-luc and ARE4-luc activity by 50%, respectively.

As shown above, AR-LBD is required for binding to YB-1. Moreover, AR ligand, DHT modulates binding of YB-1 to AR in the nucleus by promoting AR transactivation. Base on these observations, our data confirms function of YB-1 as an AR activator.

### MiR-190a-mediated suppression of AR expression and transactivation requires miR-190a binding to the 3′-UTR of *YB-1* gene

**Figure 4:**
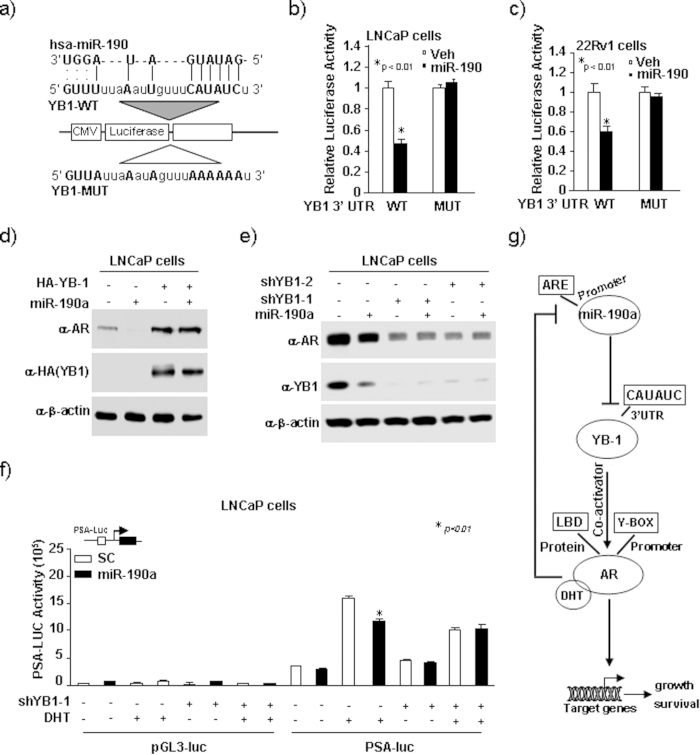
MiR-190a-mediated suppression of AR expression and transactivation requires miR-190a binding to the 3′-UTR of *YB-1* gene. (**a**) YB-1 is a potential target of miR-190a. The miR-190a targeting sites in 3′UTRs of human YB-1 are shown. (**b,c**) Luciferase/Renila activity level of 3′ UTR luciferase reporters of YB-1 in LNCaP and 22Rv1 cells at 48 hours after transfection. (**d**) LNCaP cells with stable overexpression of miR-190a and YB-1 knockdown. YB-1 and AR protein levels were determined by Western blot. (**e**) LNCaP cells with stable overexpression of miR-190a and YB-1. YB-1 and AR protein levels determined by Western blot. (**f**) LNCaP cells transiently transfected with miR-190a and/or YB-1 while treated with 10 nm DHT. PSA-Luc was assessed for AR activity. (**g**) Schematic representation of the AR/miR-190a/YB-1 negative feedback loop.

Our previous study demonstrated that miR-190a inhibited YB-1 and AR expression. In order to validate bioinformatics analysis, which indicated that only YB-1 was a potential target of miR-190a (Fig. 4a), we performed luciferase assays using reporters containing wild-type YB-1 3′-UTR and mutant defective for miR-190a binding (Fig. 4a). As shown in Fig. 4b,c, overexpression of miR-190a inhibited YB-1 wild-type, but not mutant luciferase reporter activities in LNCaP and 22Rv1 cells, indicating that miR-190a specifically targets YB-1 through direct 3′-UTR binding.

A previous study showed that YB-1 is upregulated during prostate tumor progression, and it promotes AR transcription via binding to the Y-box in the AR promoter region^10^. Our study reveals a novel function of YB-1 as AR co-activator. miR-190a inhibits YB-1 and AR expression and specifically targets YB-1 through direct 3′-UTR binding. Based on these observations, we hypothesized that miR-190a inhibits AR transactivation and expression through direct downregulation of YB-1 expression. To explore this possibility, we first overexpressed miR-190a with simultaneous depletion of YB-1 in LNCaP cells. miR-190a inhibited AR expression, while YB-1 knockdown abolished the miR-190a-mediated repression (Fig. 4d). This data suggested that miR-190a-mediated repression of AR protein levels is dependent on the presence of YB-1. Consistent with this observation, overexpression of YB-1 in LNCaP cells rescued the loss of AR expression induced by miR-190a (Fig. 4e). Luciferase reporter assays were further performed to determine whether miR-190a-mediated suppression of AR activity requires YB-1. As shown in the Fig. 4f, overexpression of miR-190a decreased androgen-induced PSA-Luc activity, while transient transfection of LNCaP cells with knockdown YB-1 abolished the repression. Taken together, these data indicate that YB-1 is required for miR-190a-mediated suppression of AR protein levels and activity (Fig. 4g).

### MiR-190a contributes to prostate cancer cell growth through AR-dependent signaling

**Figure 5:**
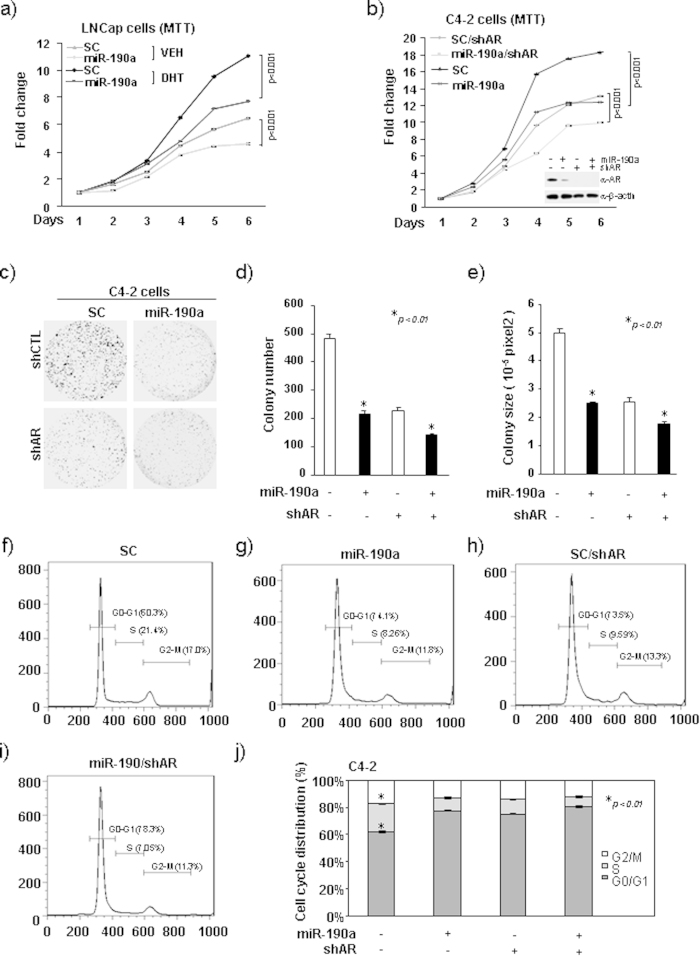
MiR-190a contributes to prostate cancer cell growth through AR-dependent signaling. (**a**) LNCaP cells with stable overexpression of miR-190a treated with or without DHT. Cells were analyzed for cell proliferation by MTT assay. (**b**) C4-2 cells with stable overexpression of miR-190a and AR knockdown. Cells were analyzed for cell proliferation by MTT assay. (**c–e**) C4-2 cells with stable overexpression of miR-190a and AR knockdown. Oncogenic growth was assessed using Colony-formation assay. Data is shown as mean ± SEM for N > 5 separate experiments. (**f–j**) C4-2 cells with stable overexpression of miR-190a and AR knockdown analyzed for cell cycle by flow cytometry. Data is shown as mean ± SEM for N > 5 separate experiments.

To determine the functional significance of miR-190a in regulating the growth of AR-positive prostate cancer cells, we conducted MTT and colony formation assays. Overexpression of miR-190a in LNCaP, C4-2 and 22Rv1 cells inhibited cell growth (Fig. 5a–e, Supplemental Fig. 2a,b).

AR has been reported to promote cell proliferation and plays a critical role in the development of prostate cancer^5^,^13^. AR agonist, DHT, stimulates castration-sensitive LNCaP cell proliferation^14^, while the knockdown of AR in castration-resistant C4-2 cells reduces cell growth^15^. To further investigate whether miR-190a-mediated inhibition of cell proliferation is dependent on AR, we overexpressed miR-190a in LNCaP cells followed by DHT treatment. Overexpression of miR-190a decreased LNCaP cell growth potential in the presence of DHT, while androgen-deprivation abolished miR-190a-mediated inhibition of cellular proliferation (Fig. 5a). Furthermore, overexpression of miR-190a with simultaneous depletion of AR in C4-2 cells inhibited cell proliferation and colony formation in the presence of endogenous AR (Fig. 5b,d), while AR knockdown alleviated the miR-190a-mediated repression. Taken together, these data suggest that AR-dependent signaling is involved in miR-190a repression of cell growth.

### MiR-190a inhibits prostate tumor growth *in vivo*

**Figure 6:**
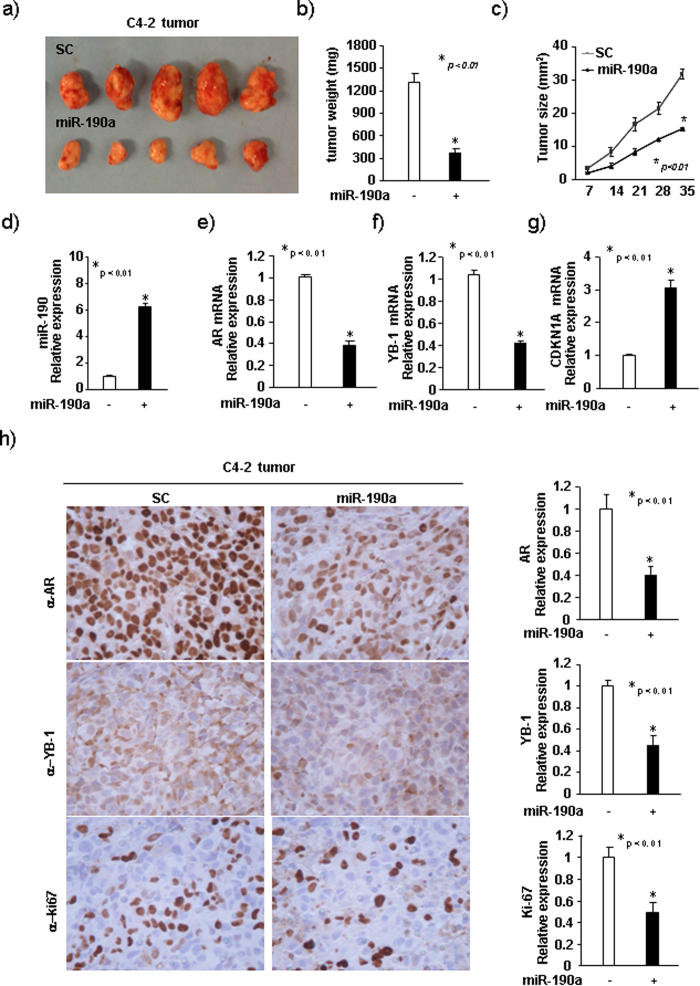
MiR-190a inhibits prostate tumor growth *in vivo*. (**a–c**) C4-2 tumors with stable overexpressed miR-190a injected into nude mice. Tumor sizes were measured every 5 days. The data is shown as mean ± SEM for N > 6 separate tumors for each group. (**a**) Images of tumors dissected from the mice. (**b**) The tumor sizes (mm3) versus days of post injection. (**c**) Tumors weights after resection at the end of experiment. (**d–g**) mRNA levels of miR-190a, AR, YB-1, CDKN1 (p21) determined by qRT-PCR from tumors. (**h**) IHC staining showing protein expression of AR, YB-1, Ki67, in C4-2 tumor tissues derived from mice. Data for quantified IHC was shown as mean ± SEM for N = 4 tumors in each group.

To investigate the role of miR-190a in inhibition of prostate cancer growth *in vivo*, we established C4-2 cells stably expressing miR-190a or miR-SC. These cells were implanted subcutaneously into immune-deficient mice and monitored for tumor growth. Overexpression of miR-190a significantly reduced both the tumor size and the tumor weight (Fig. 6a–c).

In order to determine the expression of miR-190a, AR, YB-1, CDKN1A, and Ki-67 in tumor tissues, we conducted RT-PCR and IHC staining on tumor samples. As shown in Fig. 6d–h, tumors with overexpressed miR-190a had reduced expression of AR, YB-1 and Ki-67, and increased CDKN1A, suggesting that overexpression of miR-190a inhibited prostate tumor growth *in vivo*.

### A significant negative correlation between miR-190a and AR in clinical prostate cancer specimens

**Figure 7:**
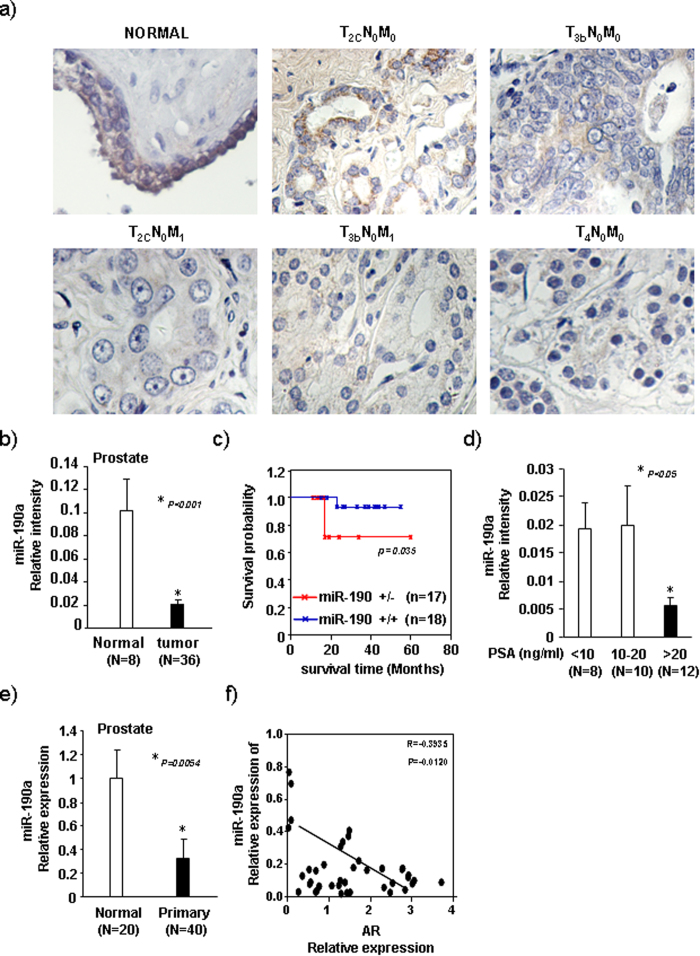
MiR-190a abundance is reduced in human prostate cancer. (**a**) Representative examples of *in situ hybridization* staining for miR-190a in each of the clinical stages of prostate cancers as indicated. (**b**) Quantification of miR-190a relative intensity for each clinical stage of prostate cancers. Data is shown as mean ± SEM for N as indicated in the figure in pair thesis as shown. (**c**) Kaplan-Meier analysis shows significant trend toward improved survival associated with high expression (n = 18) of miR-190a as opposed to low expression (n = 17) of miR-190a (p = 0.035). (**d**) *In situ hybridization* showing miR-190a expression and PSA levels in the prostate cancer specimen (n = 28). (**e**) miR-190a abundance determined by qRT-PCR. Comparison was made between normal (n = 20) and tumorous (n = 40) prostate samples. (**f**) qRT-PCR determining AR expression in the same set of prostate cancer specimens (n = 40). miR-190a and AR inverse correlation in human prostate cancers (*r* = −0.3935, *p* = 0.012).

Through *in situ* hybridization using digoxigenin-labeled locked nucleic acid (LNA)-miRNA probes we first examined miR-190a expression in 36 cases of human prostate cancer and 8 adjacent normal tissue samples obtained from tissue array. miR-190a exhibited whole cytoplasmic distribution with stronger staining in adjacent normal tissue (n = 8) when compared to prostate cancer tissues (n = 36) (Fig. 7a,b). The Kaplan-Meier analysis was conducted to evaluate the difference in disease-free survival associated with high versus low expression levels of miR-190a. The miR-190a expression was used to assign the samples to high (upper 50th percentile) or low (lower 50th percentile) groups. Patients with tumors exhibiting high miR-190a expression levels (n = 18) associated with significantly lower disease-free survival (p = 0.035) (Fig. 7c). Prostate specific antigen (PSA), a clinically relevant biomarker, is mainly induced by androgens and regulated at transcriptional level by androgen receptor (AR)^16^. We next analyzed the relationship between miR-190a and PSA. As shown in Fig. 7d, patients with high PSA levels (>20) had a lower miR-190a expression, suggesting an inverse correlation between miR-190a and AR in clinical prostate cancer specimens.

Analysis of the abundance of miR-190a and AR by Taqman quantitative real-time PCR (qRT-PCR) in prostate cancer specimens showed a significant decrease of miR-190a levels in prostate cancer tissues (n = 40) when compared to normal prostate tissue (n = 20) (Fig. 7e). miR-190a and AR were inversely correlated in human prostate cancer (r = −0.3935, p = 0.012, n = 40) (Fig. 7f), further supporting the results obtained from *in situ* hybridization.

## Discussion

Androgen receptor (AR), a member of nuclear transcription factor family, promotes tumor growth and survival-related gene transcription via androgen-mediated signaling pathways in castration-sensitive prostate cancer (CSPC)^17^. The ligand-independent activation of AR may be initiated by various biological alterations including gene mutation, gene amplification, and AR co-activator overexpression, which often leads to the failure of androgen deprivation therapy (ADT) in CRPC^18^. Blocking AR expression and activation has become one of the most effective therapies in clinical management of this disease^19^.

Prior studies have shown that a class of miRNAs plays a pivotal role in prostate cancer by acting as oncogene or tumor suppressor through androgen/AR signaling^6^,^7^. miR-205 negatively regulates AR and is associated with adverse the outcome of prostate cancer patients^20^. miR-185 suppresses proliferation, invasion and migration of human prostate cancer cells through targeting AR^21^, while the miR-124 targets AR and inhibits proliferation of prostate cancer cells^22^. Herein, we demonstrate that miR-190a regulates AR signaling through targeting YB-1, a known AR regulator. YB-1 enhances AR expression by binding to the Y-box in the promoter region of AR gene^10^. Our study reveals a novel mechanism by which miR-190a influences AR signaling.

miR-190a is known to be downregulated in aggressive neuroblastoma (NBL), and overexpression of miR-190a leads to inhibition of tumor growth and prolonged dormancy periods in fast growing tumors^8^. Recent study showed that miR-190a is involved in estrogen receptor (ERα) signaling, causing inhibition of breast tumor metastasis^9^. Androgen has been shown to repress the expression of miR-99a/let7c/125b-2 cluster through AR^23^. Little is known about whether androgen/AR signaling affects miR-190a signaling in the physiological and pathological conditions of prostate. Herein, we provided evidence from human prostate cancer tissues showing that endogenous miR-190a expression is inversely correlated with PSA levels, and that patients with higher miR-190a expression in their tumors have improved disease-free survival. PSA, as a clinically important biomarker, is mainly induced by androgen and regulated by AR at the transcriptional level^24^. Furthermore, we identified the half ARE site in the promoter region of the miR-190a gene. MiR-190a expression is repressed by AR upon androgen treatment.

YB-1 is a member of the DNA- and RNA-binding protein family with an evolutionarily ancient and conserved cold shock domain^25^, and the function is involved in a number of cellular processes including proliferation, differentiation, and stress response^26^. Extensive studies were stimulated by the identification of YB-1 as a marker of malignant cell transformation and tumor aggressiveness and as a promising molecular target to treat cancer and inflammation^25^. Prior studies focus on the shRNA targeting YB-1, however, there is little known about the endogenous miRNA that may negatively regulates YB-1. MiR-137 restored the sensitivity of the multidrug-resistant MCF-7/ADM cells to anticancer agents by targeting YB-1 in breast cancer^27^. Our study showed that miR-190a inhibited YB-1 expression. Bioinformatics analysis and 3′-UTR luciferase reporter assays demonstrated YB-1 was a direct target of miR-190a. Furthermore, miR-190a suppressed AR expression and transactivation through targeting YB-1. This study revealed a novel mechanism by which miR-190a regulates AR signaling through targeting YB-1 in the development of prostate cancer.

When AR activated by the endogenous androgenic ligands, the ligand-receptor complex translocates from cytoplasm into the nucleus, and in association with co-regulatory factors, binds to specific androgen responsive element in the regulatory regions of AR target genes^11^. AR co-regulators, including AR co-activators and co-repressors, positively and negatively regulate transcriptional activity of AR. Aberrant co-regulator function due to mutation or altered expression levels may be a contributing factor in the progression of AR-mediated diseases^28^. A previous study showed YB-1 promotes AR transcription via binding to the Y-box in the AR promoter region^10^, while we provided evidence showing YB-1 also associated with AR in the protein level. AR-LBD is required for binding YB-1. AR ligand, DHT increased the binding of YB-1 on AR in the nucleus. In addition, YB-1 promotes the AR transactivation in the presence of DHT. Base on these observations, our data indicated that YB-1 functions are as an AR activator.

The progression of prostate cancer normally advances from castration-sensitive to castration-resistant after long-term androgen deprivation therapy^2^. YB-1 functions are realted to cell proliferation, anti-apoptosis, and epithelial-mesenchymal transition in the prostate cancer^10^,^29^. YB-1 is known to be upregulated during prostate tumor progression, and promotes AR transcription via binding to the Y-box in the AR promoter region^10^. YB-1 functions as an AR co-activator. Long-term androgen deprivation induces YB-1^30^, and YB-1 overexpression converts castration-sensitive prostate cancer cells to castration-resistant cells^10^. Based on AR/miR-190a/YB-1 negative feedback loop, we speculate that, at the CSPC stage, activation of AR upon androgen binding inhibits miR-190a expression, while the androgen ablation promotes the expression of miR-190a, further causing inhibition of AR expression and activation. At the CRPC stage, long-term androgen deprivation therapy leads to abnormal activation of AR independent of androgen stimulus, which decreases miR-190a expression, further enhancing YB-1 expression and activation. This auto-regulatory negative feedback loop revealed a novel mechanism by which miRNA governs the development of prostate cancer.

In summary, we identified a biochemical and functional link between miR-190a, YB-1 and AR signaling in prostate cancer. AR/miR-190a/YB-1 signaling forms an auto-regulatory negative feedback loop in prostate cancer, where androgen/AR inhibits miR-190a expression through direct binding to the ARE in the miR-190a promoter, and miR-190a inhibits AR expression and activity through binding 3′-UTR of *YB-1* gene. In addition, YB-1 functions as a novel AR activator. Overexpression of miR-190a contributes to prostate cancer growth in CSPC and CRPC. Our results, for the first time, provide compelling evidence further rationalizing the targeting strategy to disengage the functional interaction between miR-190a and AR signaling in clinical practice to treat prostate cancer.

## Materials and Methods

### Cell culture, Plasmid construction, Reporter genes, Reagents, Expression vectors, and DNA transfection

Human prostate cancer cells LNCaP, C4-2, PC-3, DU-145, 22Rv1 and benign prostate epithelial cell line, RWPE-1, were purchased from the American Type Culture Collection (ATCC)^24^. pLe-miR-SCR lentivirus vector and pLe-miR-190a were purchased from Open Biosystems, AL, USA. pLKO lentiviral vector, pLKO-shYB1-1, pLKO-shYB1-2 were purchased from Thermo Scientific, USA. PSA-Luc and ARE4-Luc reporter genes were described^31^. miRNA luciferase reporters containing the 3′-UTR of YB-1 with miR-190a were provided by Dr. Natarajan R^32^. LNCaP, C4-2 and 22Rv1 cells infected with pLe-miR-SCR or pLe-miR-190a following by the selection with puromycin. LNCaP cells infected with pLKO -shYB1-1, pLKO -shYB1-2. GFP positive cells were selected by puromycin.

### Tissue Samples and ***In situ*** hybridization

Human prostate cancer tissue arrays were purchased from Biomax (PR956B). A collection of 40 different kinds of fresh-frozen prostate cancer tumor specimens and 20 tumor normal adjacent tissues were from Tongji Hospital, Tongji Medical College, Huazhong University of Science and Technology. All tumor samples were collected immediately after the surgical removal and snap-frozen in liquid nitrogen. Using antisense locked nucleic acid (LNA)-modified probes (Boster, Wuhan, China), *in situ* hybridization was performed as describe^33^. Oligonucleotide sequences were: LNA-miR-190a: 5′-UGAUAUGUUUGAUAUAUUAGGU-3′. These studies were carried out in accordance with the approved guidelines. All the experimental protocols were approved by the Institutional Review Board of the Tongji Hospital, Tongji Medical College, Huazhong University of Science and Technology.

### RNA Isolation and Real-time PCR analysis

Total RNAs were extracted with Trizol reagent according to the manufacturer’s instruction (Invitrogen, CA, USA). To determine the mRNA levels of AR, YB-1 and PSA, total RNAs were reversely transcribed by iScript cDNA Synthesis Kit (Bio-Rad Laboratories, Hercules, CA), respectively, according to the manufacturer’s instructions^34^. The 18 S rRNA gene was used as reference for normalization^34^. Taqman RT-qPCR was performed to detect mature miRNA expression using Taqman miRNA reverse transcription kit, has-miR-190a (AB Assay ID: 000489) and RNU6B (U6, AB Assay ID: 001093) according to the manufacturer’s protocol (Applied Biosystems)^35^. The U6 was used as reference for normalization^35^.

### Cell proliferation assay

Cells infected with miR-190a and miR-SCR were seeded into 96 well plates in normal growth medium, and cell growth was measured daily by MTT assays using 3-(4, 5-dimethylthiazol-2-yl)-2, 5-diphenyltetrazolium bromide.

### Colony Formation Assays

Cells were plated in triplicates in 3 ml of 0.3% agarose (sea plaque) in complete growth medium overlaid on 0.5% agarose base, also in complete growth medium. Two weeks post cell seeding, colonies were visualized after staining with 0.04% crystal violet in methanol for 1–2 hrs. The colonies more than 50 μm in diameter were counted using an Omnicon 3600 image analysis system.

### Luciferase Assays

Cells were seeded at a density of 1 × 10^5^ cells in a 24-well cell culture plate on the day prior to transfection with Superfect according to the manufacturer’s protocol (Qiagen, Valencia, CA). For reporter gene assays, a dose-response was determined in each experiment with 50 and 200 ng of expression vector and promoter reporter plasmids (0.5 μg). Luciferase activity was normalized for transfection efficiency using *β*-galactosidase reporter as an internal control. The fold effect of expression vector was determined with comparison to the value of the empty expression vector cassette and statistical analyses were performed using the t-test^35^,^36^.

### Immunoprecipitation and Western blot

Immunoprecipitation (IP) and Western blot assays were conducted in LNCaP, C4-2, 22Rv1 and PC3 and 293 T cells as indicated. Cells were pelleted and lysed in buffer (50 mmol/L HEPES, pH7.2, 150 mmol/L NaCl, 1 mmol/L EDTA, 1 mmol/L EGTA, 1 mmol/L DTT, and 0.1% Tween 20) supplemented with a proteaseinhibitor cocktail (Roche Diagnostics). Antibodies used for Western Blot Analysis. IP and Western blot were: AR (H280) (SC-13062, Santa Cruz), YB-1(SC-101198, Santa Cruz), YB-1(2749 S, Cell Signaling), c-myc (SC-40, Santa Cruz), c-myc (SC-788, Santa Cruz), anti-Gal4 (SC-577, Santa Cruz) and PSA (SC-7316, Santa Cruz).

### ChIP Analysis

ChIP assays were performed according to the protocol of the Upstate Biotechnology as described (31). The primer sequence for miR-190a promoter is are P1(−1 ~ −168): 3′CTGGTGCATGTGCTGACAGT-5′, 3′-GAAAAGGCATCCAGGTTTGA-5′; P2(−229 ~ −395): 3′-TCAGGAAGAGTTTGGGGAGA-5′, 3′-ATCCCAGGCAAAAAGTGATG-5; P3(−411 ~ −584): 3′-GCACCAAAATCAGCCAGTCT-5′. 3′-CCCCAGAAGGAACACACATC-5′; P4(−684 ~ −820): 3′-CCATCGTATTAGGAAGGGTGA-5′, 3′-TGCCCTATTAGGCACAAAAA-5′; P5(−834 ~ −988): 3′-GCTGAAGTAGGCCTGTGAGG-5′, 3′-AGGAACCCTCAAACAAAGGA-5′. Polyclonal antibody to AR (SC-13062, Santa Cruz) was used for IP and normal IgG was used as a negative control. One tenth of original DNA was used as an input control.

### Cancer micro-RNA Array assays

Cancer micro-RNA Array assays were conducted as described^37^,^38^. Differential expression of 95 miRNAs was analyzed by RT-PCR using the QuantiMir System (SBI System Biosciences). All 95 miRNAs chosen for the array are based on their potential roles in cancer, cell development and apoptosis. cDNAs from different cell lines were mixed with SYBR® Green Mastermix (Bio-Rad Laboratories, Hercules, CA) plus the universal reverse primer. Specific primers (1 μl) were added each well of the qPCR plate. Expression levels of each mature miRNA were evaluated using comparative threshold cycle (Ct) method as normalized to that of U6 (2^−ΔCt^). The array plate also included the U6 transcript as a normalization signal.

### *In vivo* tumor implantation

C4-2 cells stably expressing miR-190a and miR-SCR were injected subcutaneously into 4-6-week-old castrated mal nude mice purchased from Beijing HFK Bio-Technology.co., LTD. Tumor growth was measured using a digital caliper every 5 days for 4–5 weeks. Tumor weight was measured when mice were sacrificed on day 32 after cell implantation. Immunohistochemical staining under the standard procedure as described before.

These studies were carried out in accordance with the guidelines of the Tongji Hospital Institute for Animal Studies. All the experimental protocols were approved by the Institutional Animal Care and Use Committee at Tongji Hospital, Tongji Medical College, Huazhong University of Science and Technology.

### Statistical analysis

All statistical analyses were performed using EXCEL 2010 (Microsoft, USA). All *in vitro* experiments were performed in triplicate, and all data were represented as mean ± SD. Statistical analyses were conducted using Student’s t test and Pearson Correlation Coefficient. The significance of each value was determined when P value was less than 0.05.

## Additional Information

**How to cite this article**: Xu, S. *et al.* The inhibitory effects of AR/miR-190a/YB-1 negative feedback loop on prostate cancer and underlying mechanism. *Sci. Rep.* **5**, 13528; doi: 10.1038/srep13528 (2015).

## Supplementary Material

Supplementary Information
