# Supplementary material for: The inhibitory effects of AR/miR-190a/YB-1 negative feedback loop on prostate cancer and underlying mechanism
Source: Sci Rep. 2015 Aug 28;5:13528. doi: 10.1038/srep13528 (PMC4551971; doi:10.1038/srep13528)
Supplement: Supplementary Information [file srep13528-s1.doc]

**The inhibitory effects of AR/miR-190a/YB-1 negative feedback loop on prostate cancer and underlying mechanism**

Shaohua Xu^1^, Tao Wang^2^, Wen Song^2^, Tao Jiang^4^, Feng Zhang^5^, Yu Yin^6^, Shi-Wen Jiang^7^, Kongming Wu^8^, Zuoren Yu^9^, Chenguang Wang^10^, Ke Chen^2^, ^3, *^

**SUPPLEMENTAL DATA**

**Supplemental Figure 1. MiR-190 and AR expression in prostate cell lines** (a-b) qRT-PCR analysis of miR-190 expression in prostate cell lines (RWPE1, LNCaP, LAPC4, C4-2, 22Rv1, DU145, PC3).

**Supplemental Figure 2. Mir-190 inhibits cell proliferation in 22RV1 cells.** (a) 22Rv1 cells with stable overexpression of miRNA-190. Cells were analyzed for cell proliferation by MTT assay. (b) 22Rv1 cells with stable overexpression of miR-190. Oncogenic growth by Colony-forming assay. Data is shown as mean ± SEM for N > 5 separate experiments.
